# Muscle Non-shivering Thermogenesis and Its Role in the Evolution of Endothermy

**DOI:** 10.3389/fphys.2017.00889

**Published:** 2017-11-09

**Authors:** Julia Nowack, Sylvain Giroud, Walter Arnold, Thomas Ruf

**Affiliations:** Department of Integrative Biology and Evolution, Research Institute of Wildlife Ecology, University of Veterinary Medicine, Vienna, Austria

**Keywords:** brown adipose tissue, cold exposure, non-shivering thermogenesis, SERCA, sarcolipin, skeletal muscle, UCP1

## Abstract

The development of sustained, long-term endothermy was one of the major transitions in the evolution of vertebrates. Thermogenesis in endotherms does not only occur via shivering or activity, but also via non-shivering thermogenesis (NST). Mammalian NST is mediated by the uncoupling protein 1 in the brown adipose tissue (BAT) and possibly involves an additional mechanism of NST in skeletal muscle. This alternative mechanism is based on Ca^2+^-slippage by a sarcoplasmatic reticulum Ca^2+^-ATPase (SERCA) and is controlled by the protein sarcolipin. The existence of muscle based NST has been discussed for a long time and is likely present in all mammals. However, its importance for thermoregulation was demonstrated only recently in mice. Interestingly, birds, which have evolved from a different reptilian lineage than mammals and lack UCP1-mediated NST, also exhibit muscle based NST under the involvement of SERCA, though likely without the participation of sarcolipin. In this review we summarize the current knowledge on muscle NST and discuss the efficiency of muscle NST and BAT in the context of the hypothesis that muscle NST could have been the earliest mechanism of heat generation during cold exposure in vertebrates that ultimately enabled the evolution of endothermy. We suggest that the evolution of BAT in addition to muscle NST was related to heterothermy being predominant among early endothermic mammals. Furthermore, we argue that, in contrast to small mammals, muscle NST is sufficient to maintain high body temperature in birds, which have enhanced capacities to fuel muscle NST by high rates of fatty acid import.

## Introduction

The evolution of endothermy is of major interest in the understanding of mammalian and avian radiation. It is often debated when and how the transition from ectothermic reptiles to endothermic mammals and birds occurred. In terms of the underlying ultimate factors leading to the evolution of endothermy, there are currently two dominating hypotheses: The “increased levels of activity” or “aerobic capacity” model (Bennett and Ruben, 1979) and the “parental care” model (Koteja, 2000). In essence, the aerobic capacity model postulates that maximum metabolic rate, a proxy of aerobic capacity and sustained activity, is the target of directional selection. In this model, elevations in basal metabolic rate (BMR) are only a correlated consequence of increased maximum metabolism. The parental care model, on the other hand, assumes that increased investment into offspring required increased rates of energy assimilation, which led to enhanced function and metabolism of visceral organs. In both models increased aerobic tissue metabolism is accompanied by increased mitochondrial density, increased mitochondrial membrane surface, and elevated enzyme activities (Hulbert and Else, 2000). It has also been proposed that BMR was elevated by increased membrane leakiness caused by the incorporation of polyunsaturated fatty acids (PUFA) (Hulbert and Else, 1999, 2000; Hulbert, 2003, 2005), a view that has been challenged by a comparative study on mammals (Valencak and Ruf, 2007). However, there still may be direct effects of certain PUFA on membrane-bound enzymes that may well affect e.g., seasonal adjustments of metabolism (reviewed in Arnold et al., 2015). While both, birds and mammals, can defend their body temperature (T_b_) within the thermoneutral zone by basal metabolism based on the above described processes, the biochemical basis of heat production during cold exposure seems to differ in both groups. Hence, most researchers in the field assume that endothermy among vertebrates was developed twice: once within the bird lineage and once within mammals. A commonly accepted view is that small placental mammals were able to colonize colder habitats because they are able to maintain high T_b_ even in the cold by producing heat via non-shivering thermogenesis (NST) mediated by the uncoupling protein 1 (UCP1) in brown adipose tissue (BAT) (Chaffee et al., 1975; Foster and Frydman, 1978; Heaton et al., 1978). Birds, on the other hand, lack this mechanism (Emre et al., 2007) and seem to rely on shivering and non-shivering heat production in muscle during cold exposure (Dawson and Carey, 1976; Bicudo et al., 2001). However, BAT and functional UCP1 are not present in all mammalian species. Marsupials, monotremes (Jastroch et al., 2008; Polymeropoulos et al., 2012), and certain placental mammals lack functional BAT (Gaudry et al., 2016). A recent study has shown that mutations inactivating UCP1 have occurred in at least eight of the 18 placental mammal orders (Gaudry et al., 2016), questioning the importance of BAT-mediated NST as the key thermoregulatory component in all placental mammals. The existence of a mechanism of muscular NST has long been suspected, i.e., for marsupials and monotremes (e.g., Nicol et al., 1992; Grigg et al., 2004). While the principle mechanism of uncoupled NST via sarcoplasmatic reticulum Ca^2+^-ATPase (SERCA) activity was studied and described extensively in rabbits by de Meis et al. (e.g., de Meis, 2001a; de Meis et al., 2005b), the precise mechanism, i.e., the role of sarcolipin (SLN), and its importance for thermoregulation was only recently discovered (Bal et al., 2012, 2016). Importantly, Rowland et al. (2014) suggested that NST in skeletal muscle—which can occur independently of shivering—was the earliest facultative thermogenic mechanism in vertebrates, before evolutionary pressure resulted in the development of a mechanism (UCP1 in BAT) allowing for higher rates of heat production without interference with muscle function. Muscle NST may have evolved earlier than classical UCP1-dependent BAT thermogenesis, which is not a characteristic trait of all endotherms and the transition from ectothermy to endothermy did not depend on BAT. In this review we summarize our current knowledge on muscle-based NST, from here on referred to as muscle NST to distinguish it from UCP1-mediated NST in BAT, and add more evidence to the hypothesis that muscle NST could have been the earliest mechanism of endogenous heat production in vertebrates. We also discuss hypotheses why small placental mammals, despite the existence of muscle NST, additionally evolved UCP1-mediated NST in BAT, and why birds did not.

## Non-shivering thermogenesis in muscle: how does it work?

The “classical” mechanism of NST in BAT involves the protein UCP1 that facilitates proton leakage across the inner mitochondrial membrane, leading to futile cycling of protons and to heat generation instead of adenosine triphosphate (ATP) production (Nedergaard and Cannon, 1985). Another mechanism of heat production in mammals involves Ca^2+^-slippage in skeletal muscle cells (myocytes). A seminal study on knockout mice has shown that this form of NST is crucial in supporting the maintenance of high T_b_ in absence of BAT-mediated NST, and that it is controlled by the protein SLN (Bal et al., 2012). The mechanism of this muscle NST is based on activity of a Ca^2+^-ATPase, i.e., SERCA, in the sarcoplasmatic reticulum (SR). During muscle contractions SERCA removes Ca^2+^ from the myocyte cytosol (Hasselbach and Makinose, 1961, 1963; Periasamy and Huke, 2001) back into the SR, thereby triggering muscle relaxation before the initiation of the next contraction phase. However, SERCA does not always use the entire energy derived from ATP-hydrolysis to pump Ca^2+^-ions across the SR membrane, a variable part (between ~10 and 25 kcal/mol ATP) is released as heat (de Meis, 2002; de Meis et al., 2005b). The partitioning is regulated by the gradient between cytosolic and SR luminal concentrations of Ca^2+^ and the protein SLN (Asahi et al., 2003; Mall et al., 2006). High luminal concentrations of Ca^2+^ uncouple ATP-hydrolysis from Ca^2+^ transport across the SR membrane by causing the release of the two Ca^2+^-ions bound to SERCA back to the cytoplasmic side of the membrane rather than to the luminal side (reviewed in Mall et al., 2006). Such “slippage” creates an uncoupling of SERCA activity from Ca^2+^ transport into the SR, i.e., no actual transport of Ca^2+^-ions and converts the energy from ATP-hydrolysis into heat (Asahi et al., 2003; Maurya et al., 2015). This process is fostered by SLN although it is not yet possible to explain the effect of SLN on slippage in molecular terms (Mall et al., 2006). In short, SLN allows ATP hydrolysis to occur but interferes with calcium transport, resulting in the release of calcium back into the cytosol (de Meis, 2001b). This leads to two effects: First heat is produced by SERCA and second, SLN maintains high Ca^2+^ levels in the cytosol, hence activating Ca^2+^-dependent pathways that regulate muscle metabolism and mitochondrial activity (Sahoo et al., 2013).

SERCA is expressed in seven different isoforms in mammalian tissues. The most likely ones involved in thermogenesis, due to their expression in skeletal muscle, are SERCA1a (mainly in fast twitch fibers) and possibly SERCA2a (in slow twitch and fast-oxidative fibers) (Periasamy and Kalyanasundaram, 2007). Muscle NST has been mainly studied in SERCA1a, but there is evidence that SERCA2a can also modulate the amount of heat produced during ATP hydrolysis (reviewed in Pant et al., 2016). Another regulator of SERCA, the protein phospholamban, is not involved in thermogenesis (Sahoo et al., 2013; Shaikh et al., 2016). Phospholamban affects the apparent affinity of SERCA for Ca^2+^ but does not affect the maximal velocity (V_max_) of SERCA Ca^2+^ uptake into SR whereas SLN decreases the V_max_ of SERCA Ca^2+^ transport into SR but does not affect the affinity of SERCA for Ca^2+^, i.e., can even bind to SERCA at high concentrations of Ca^2+^. In other words, phospholamban acts as a brake on SERCA activity until it is dissociated by either phosphorylation or by high Ca^2+^ (Shaikh et al., 2016), whereas SLN facilitates heat production by SERCA. Recently, a third regulator of SERCA, myoregulin, has been identified, but to date its role is not well-understood (Anderson et al., 2015). Interestingly, SERCA can also be regulated by the concentration of certain PUFA in the surrounding SR membrane, with very large effects on SERCA activity (Swanson et al., 1989). This may explain the effects of certain dietary PUFA on hibernation (Ruf and Arnold, 2008). For instance, a study on hibernating Syrian hamsters (*Mesocricetus auratus*) has shown that cardiac SERCA activity was enhanced by high n-6 PUFA content in SR phospholipids, allowing them to reach lower T_b_, but depressed by high amounts of n-3 PUFA (Giroud et al., 2013). Details on the specific effects of PUFA are reviewed elsewhere (Arnold et al., 2015).

**Figure 1:**
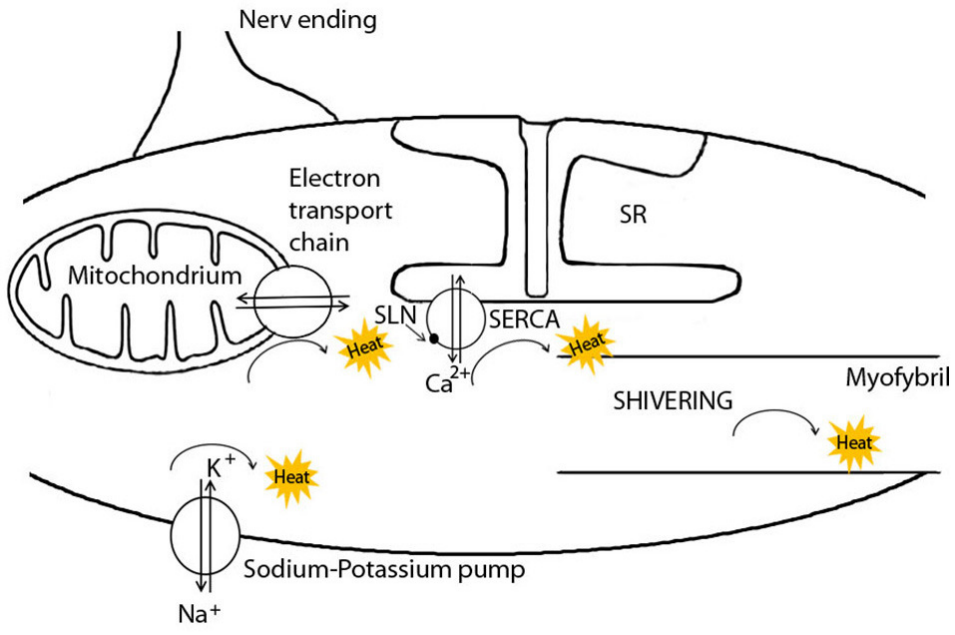
The pathways of heat generation in muscle cells. Non-shivering thermogenesis (NST) in muscle cells is activated by the binding of the peptide sarcolipin (SLN) to the Ca^2+^ ATPase (SERCA), the transmembrane Ca^2+^ pump located in the sarcoplasmatic reticulum (SR) membrane. SLN causes Ca^2+^-slippage with the sole purpose of heat generation. Heat is also generated by mitochondria, the sodium-potassium pump, and myofibril contraction. For more details see text. Modified after Herpin et al. (2002).

NST via SERCA is of course only one of several different pathways of heat production in skeletal muscle cells (Figure 1). First, heat is generated in mitochondria during ATP synthesis since some protons always leak through the inner mitochondrial membrane, rather than through the ATP synthase (Rolfe and Brand, 1997; Clarke et al., 2013). Secondly, heat is generated during ATP hydrolysis in several enzymatic reactions, when the energy released exceeds that required to drive a reaction. This is the case for the ATP utilization by the sodium-potassium pump (Na^+^-K^+^-ATPase), the myosin-ATPase during muscular work or shivering, and by SERCA. SERCA activity produces up to 25% of the metabolic rate of a resting muscle (Simonides et al., 2001) and thus contributes to elevations of BMR when muscles are enlarged in response to cold (e.g., Vézina et al., 2017). Further heat will be generated by SERCA during both shivering, when Ca^2+^ pumping is coupled to myofibril contraction and when it is uncoupled, i.e., when SERCA serves as a heat generator by slippage of Ca^2+^-ions. In this case, ATP is cleaved without apparent work and then the ADP produced is phosphorylated by the mitochondria, leading to an increase in oxygen consumption (de Meis, 2001b). Hence mitochondrial oxidative phosphorylation also contributes to muscle-NST. All of these pathways of heat generation may be increased in response to cold exposure, albeit on different time scales. For instance, in cold-adapted rats, the total volume of mitochondria was significantly increased by 37% in the musculus soleus after 3 weeks, increasing the capacity for heat production during oxidation of fuels and ATP synthesis (Buser et al., 1982). Similarly, the total activity of the Na^+^-K^+^-ATPase can be significantly up-regulated during cold exposure in pigs (Herpin et al., 1987). It seems, however, that this up-regulation is based on the relatively slow process of increasing the enzyme's expression level (Clarke et al., 2013), or changing the fatty acid composition or cholesterol content of the surrounding membrane (Cornelius, 2001). In contrast, the activation of heat generation upon cold exposure by SERCA via SLN should be instantaneous (Bal et al., 2012). Indeed, Suzuki et al. (2007) could demonstrate an increase of heat production in single cells within seconds after experimentally causing influx of extracellular Ca^2+^ (Suzuki et al., 2007). This increase in heat production was suppressed when SERCA activity was specifically blocked (Suzuki et al., 2007). Thus, apart from myofibril contraction, ATP hydrolysis by SERCA apparently is the only mechanism in muscle that can be immediately up-regulated in response to cold.

Heat producing mechanisms that involve SERCA are also known from birds and fishes. In birds there is a profound increase in SERCA activity during cold exposure-similar to muscle NST in mammals (Dumonteil et al., 1993, 1995). Interestingly, SERCA2a remains largely unchanged, whereas SERCA1a increases its level of expression with prolonged acclimatization (Dumonteil et al., 1995). This suggests that in birds, SERCA2a may be primarily involved in shivering thermogenesis in slow-twitch fibres, whereas SERCA1a seems responsible for ATP cleavage during muscle NST in fast-twitch fibers (Dumonteil et al., 1995). In birds, prolonged cold exposure also leads to a 30–50% increase in ryanodine receptors (RyR) (Dumonteil et al., 1995), i.e., the Ca^2+^ channels through which Ca^2+^ is normally released from the SR. Presently it is unclear whether muscle NST in birds also involves Ca^2+^ slippage controlled by SLN. However, the involvement of SLN in muscle NST in birds seem unlikely, since the C-terminus, which appears crucial for the regulation of SERCA (Barbot et al., 2016), has a differing amino acid-sequence (KSYQE/Q instead of RSYQY) (Montigny et al., 2014). Interestingly, a study on ducklings found that muscle NST was correlated to changes in avian UCP, a paralog of the mammalian UCP1, (the UCP1-locus has been lost in birds, Emre et al., 2007), which—in contrast to mammalian UCP1—is not associated with a change in mitochondrial membrane conductance, but involved in muscle thermogenesis. The exact mechanisms are still unclear (Teulier et al., 2010), however, avian UCP expression is restricted to skeletal muscle and its abundance increases under cold-acclimatization (Raimbault et al., 2001).

A form of muscle NST has also at least evolved twice in fishes, as it is found in certain fish species that show regional endothermy like billfish and butterfly mackerel (Block, 1994). The so-called “heater organ,” a specialized tissue next to the eyes, derived from muscle, uses futile Ca^2+^ cycling to raise the local temperature by some degrees above that of the surrounding water (in the case of the swordfish up to 15°C), thereby enhancing temporal resolution of vision (Carey, 1982; Fritsches et al., 2005). Although derived from extraocular muscle fibers, cells of the heater organ have lost most of the contractile myofilaments that are characteristic for muscle tissue. Instead, the cells express a modified muscle phenotype with a high mitochondrial and SR content. The latter is enriched in SERCA1a pumps and RyR. These Ca^2+^ channels cause a release of sequestered Ca^2+^ from the SR, whereas SERCA1a pumps it back into the organelle, leading to futile Ca^2+^ cycling and heat production (Block et al., 1994; Morrissette et al., 2003; da Costa and Landeira-Fernandez, 2009). Taken together it seems that the mechanism of muscle NST in mammals, birds and even fish involves ATP hydrolysis by SERCA. However, although SLN is already found in fishes and reptiles (Newman et al., 2013), it is so far not known if it is involved in heat production in these taxa. If this is not the case, the SLN-induced slippage of Ca^2+^-ions without involvement of the RyR (Mitidieri and de Meis, 1999; Mall et al., 2006) would be an alternative form of thermogenesis restricted to mammals. But at this point, this remains speculation.

Another organ that seems to benefit from local heating is the heart, for which maintaining functionality even at low T_b_ is most important. SERCA2a is the major isoform in the heart and is primarly involved in Ca^2+^ handling to ensure proper cardiac function. The capability of up-regulating SERCA2a activity, already present in fish, could be crucial for the maintenance of high heart rates and, as a secondary function, also for regional endothermy and therefore could have contributed to the evolution of endothermy and the colonization of cold habitats. Interestingly, gene-expression and protein levels of SERCA2a are increased in the hearts of hibernators in winter compared with those in the non-hibernating season (Yatani et al., 2004; Brauch et al., 2005): A higher density of SERCA2a in the SR membranes accelerates Ca^2+^-uptake, an adaptation needed to counteract the temperature-dependent (Arrhenius) effect on maximum SERCA activity at low T_b_ during torpor. The high density of SERCA and the concomitant high amount of hydrolysis of ATP also provides the potential for muscle NST (de Meis, 2002; Andrews, 2007). More evidence for this secondary role of SERCA2a in the heart was collected by Ketzer et al. (2009), who evaluated the contribution of cardiac tissue in rabbits by measuring mitochondrial respiration in permeabilized cardiac muscle and specifically looked into heat produced by Ca^2+^ transport (Ketzer et al., 2009). They found an increase in oxygen consumption and associated heat production during Ca^2+^ transport by cardiac SR after short-term cold exposure. The extra heat produced by the heart under these conditions was mainly derived from both an increase of SERCA2a activity and an enhancement of mitochondrial oxidative phosphorylation. These data suggest that heat production through SERCA2a in cardiac muscles leads to regional endothermy, helping the heart to sustain proper contractions and work load. However, there is no evidence that this local heat production involves SLN and in fact SLN is not expressed in the ventricles of small rodents (Vangheluwe et al., 2005).

## Importance of muscle NST in mammals

Muscle NST in mammals has been known to exist for decades and the biochemical mechanisms involved in muscle NST were studied extensively in the past (e.g., Clausen et al., 1991; Mitidieri and de Meis, 1999; de Meis, 2001b). However this type of NST has been shown only recently to represent an essential source of endogenous heat production that allows mammals to remain euthermic in the cold (Bal et al., 2012), and therefore data on muscle NST in mammals are still scarce (see Table 1). Although only clearly confirmed in laboratory strains of mice and rats—species that usually possess functional BAT (Babu et al., 2007; Bal et al., 2012; Pant et al., 2015)—skeletal muscles meet all relevant preconditions to be the site of a ubiquitous heat production mechanism in all endotherms. Skeletal muscle represent the largest fraction of body mass in mammals and birds, and interestingly, are about 30% more massive in mammals than in similar-sized ectothermic reptiles (Ruben, 1995; Rowland et al., 2014). Even more support for the ubiquitous involvement of muscle NST in thermogenesis of mammals comes from studying hibernation. Not all mammals maintain a high T_b_ throughout the year. So called heterothermic mammals often reduce their energetic demands during challenging periods by using short bouts of torpor or months long hibernation, both characterized by a tremendous reduction of metabolic rate, endogenous heat production and therefore T_b_, and inactivity. It has recently been shown that SERCA1a and SLN are significantly reduced during the hibernation season in skeletal muscles of thirteen-lined ground squirrels [*Ictidomys* (formely *Spermophilus) tridecemlineatus*] (Anderson, 2016; Anderson et al., 2016). While this downregulation could be due to reduced muscle function during inactivity, it is surprising that SLN, the regulator of ATP hydrolysis efficiency of SERCA, i.e., the regulator of the amount of heat produced, is also reduced. This could suggest that the reduced expression of SLN is rather correlated to an actively down-regulation of metabolic rate to save energy, which in turn would indicate that muscle NST plays an important role in the thermogenesis and energy expenditure of ground squirrels.

**Table 1:** Evidence of muscle-based non-shivering thermogenesis in vertebrates.

**Taxon**	**Species**	**Evidence for muscle NST**	**References**
Fish	Billfish, butterfly mackerel	No BAT, no thermogenic function of UCP1. Heater organ- release of sequestered Ca^2+^ from the SR via ryanodine receptors. SERCA1a pumps it back into the organelle, leading to Ca^2+^ cycling and heat production.	Block, 1994; Jastroch et al., 2005
Amphibia	NA	Not yet investigated.	
Reptilia	Potentially tegu lizards	No BAT, no thermogenic function of UCP1. Tegu lizards maintain a T_b_ of 5–6°C above ambient during the reproductive season and even during the colder night hours, when an increase of T_b_ via basking is not possible.	Tattersall et al., 2016
Birds	Several species	No BAT, no UCP1. A release of sequestered Ca^2+^ from the SR via ryanodine receptors. SERCA1a pumps it back into the organelle, leading to Ca^2+^ cycling and heat production.	Dumonteil et al., 1995
Mammals			
Monotremata	Anecdotal evidence for echidnas	No BAT, no evidence for thermogenic UCP1; Speculations about alternative rewarming mechanism from torpid states.	e.g., Grigg et al., 1992, 2004; Nicol et al., 1992
Marsupialia	NA	No BAT, no thermogenic function of UCP1; speculations about alternative rewarming mechanism from torpid states.	e.g., Nicol et al., 1992; Grigg et al., 2004
Placentalia	Found in rodents, lagomorpha; strong evidence for pigs	BAT and thermogenic function of UCP1 in most species; Evidence of sarcolipin-regulated muscle NST in mice and rats (in addition to UCP1/BAT); Downregulated sarcolipin gene expression in thirteen-lined ground squirrels. Muscle NST via SERCA found in rabbits; Likely in piglets- increasing thermogenic capacity in piglets, while at the same time shivering is decreasing.	Berthon et al., 1994, 1996; de Meis, 2001a,b; de Meis et al., 2005b; Babu et al., 2007; Bal et al., 2012, 2016; Anderson, 2016; Anderson et al., 2016; Pant et al., 2016

Although SLN is only expressed in amounts likely to small to have a measurable effect on thermogenesis in mice with intact BAT/UCP1 (Butler et al., 2015), SLN has been proven to be important for maintenance of endothermy when NST in BAT is not possible (Bal et al., 2012). Interestingly, both mechanisms of NST—UCP1-mediated as well as muscle NST—can compensate for the loss of one system, while double-knockout mice without UCP1 and SLN are unable to survive during prolonged cold exposure, indicating that at least one of the two mechanisms is pivotal to maintain endothermy (Rowland et al., 2015). Furthermore, studies on the significance of muscle NST in UCP1-knockout mice, as well as BAT-ablated mice have shown that the efficiency of muscle NST can be increased with long-term exposure to mild cold (4°C), while shivering thermogenesis is reduced (Rowland et al., 2015; Bal et al., 2016). It is generally assumed that BAT is the principal site of NST in small cold-adapted mammals, but this notion is challenged by the finding of compensation of UCP1/BAT-dysfunction by muscle NST, although it is questionable whether UCP1-knockout mice can actually maintain their T_b_ at ambient temperatures below 4°C. Furthermore, a recent study has shown that UCP1-inactivating mutations have occurred in at least eight of the 18 placental mammalian orders, mainly larger-bodied species (Gaudry et al., 2016), suggesting that at least large species do not depend on UCP1-mediated NST in BAT.

A commonly shared view suggests that the thermogenic evolution of UCP1 has occurred after the divergence between placentals and marsupials (Saito et al., 2008). Interestingly, UCP1 orthologs have been identified in non-placental mammals, as well as in fish (Jastroch et al., 2005, 2008), but UCP1 has a unique function in placentals in that it is necessary for H^+^-driven NST in BAT (Hughes et al., 2009). This is consistent with the fact that all studies looking into UCP1-mediated NST and the presence of BAT in marsupials and monotremes, which diverged from placental and marsupials even earlier, so far failed to find clear evidence for UCP1-mediated NST (Nicol, 1978; McNab and Wright, 1987; Hayward and Lisson, 1992; Nicol et al., 1997; Opazo et al., 1999; Rose et al., 1999; Kabat et al., 2003; but see: Polymeropoulos et al., 2012). Although the mechanism of NST in marsupials and monotremes remains elusive, it has often been speculated that an UCP1-independent mechanism of NST must exists in both groups (e.g., Nicol et al., 1992; Grigg et al., 2004). Anecdotal evidence of a hibernating echidna retrieved from its hibernaculum at a T_b_ of about 13°C showed that the individual rewarmed to about 18°C without any visible signs of shivering or muscular movement except for occasional very slow movements of the limbs and body, before body twitches and shivering were observed above 18°C (Grigg et al., 1992). On the first glance the idea that muscle NST might be important for rewarming from torpor contradicts the earlier finding of low SLN gene expression throughout the hibernation season in ground squirrels (Anderson, 2016). However, ground squirrels have functional UCP1 and BAT and therefore no need to rely on muscle NST during arousals. Interestingly, monotremes and marsupials, which, although inhabiting generally warmer areas, can also be found in habitats with temperate climate and coldish winter temperatures, have lower resting T_b_ than placental mammals. Monotremes consist of the aquatic platypus (*Ornithorhynchus anatinus*) and terrestrial echidnas (*Tachyglossidae*). The short-beaked echidna (*Tachyglossus aculeatus*), which is the only of the echidna species we have sufficient knowledge on, has a modal T_b_ of about 32°C with daily amplitudes of 2–5°C and an often labile T_b_ that rises as a result of activity and declines during inactivity (Grigg et al., 2004). Furthermore, echidnas often enter torpid states, thereby allowing their T_b_ to drop to levels as low as ambient. Resting T_b_s of small-sized marsupials are ranging between 32 and 35°C (Geiser, 2004) and most small-bodied marsupials are known to regularly enter torpor (Geiser and Körtner, 2010). Furthermore, some marsupials, such as antechinus (a very small, nocturnal mouse-like marsupial), are highly susceptible to develop hypothermia when cold stressed, although they are able to undergo torpor and regulate the decrease in T_b_ (Geiser, 1988). Thus, it seems that in the case of very small species the lack of BAT may indeed be associated with increased difficulties dealing with cold conditions.

## If muscle NST was sufficient, why did UCP1-mediated thermogenesis in bat evolve?

If the anecdotal reference by Grigg et al. (1992) reported above is supported by future studies demonstrating muscle NST in monotrenes or marsupials, this would mean that NST in skeletal muscle is more ancient than NST in BAT. The existence of two mechanisms of NST, a likely more ancient mechanism in muscle and the later evolved mechanism of short-circuiting the proton gradient in BAT, leads to the question about the selective advantage associated with the latter. Speculations about the ultimate reasons for the evolution of UCP1 mediated thermogenesis in BAT include various scenarios: In addition to the hypothesis that this mechanism enabled animals to colonize colder habitats that we already mentioned above, speculations include (1) defense against the natural cold stress of birth (Cannon and Nedergaard, 2004), (2) enabling a high T_b_ for periods of parental care (Oelkrug et al., 2013), (3) incompatibility of locomotor performance and muscle NST (Rowland et al., 2014), and (4) rapid arousal from torpor as well as decreasing the energetic costs of rewarming from torpor (Oelkrug et al., 2011). Below, we reevaluate these hypotheses in the light of the existence of muscle NST.

It has been hypothesized that UCP1 mediated thermogenesis in BAT evolved as a defense of the cold stress of birth (Cannon and Nedergaard, 2004), when mammals leave the warm body of the mother and have to cope with considerably lower outside temperatures. Could muscle NST be not sufficient for thermoregulatory demands of neonates? Even in larger species, neonates have high demands of thermogenesis. They lose more heat than the bigger adults because of their large surface area to volume ratio and have less insulation. Interestingly, SLN expression is high in newborn mice and rats - two species that have muscle as well as UCP1-mediated NST - and is usually down-regulated during neonatal development (Babu et al., 2007; Pant et al., 2015); however, continuous cold exposure can prevent this down-regulation, leading to an increased thermogenic capacity (Pant et al., 2015). Another interesting taxon in this context are pigs. Both wild and domestic pigs lack BAT (Trayhurn et al., 1989) and the UCP1-mediated NST capacity (Berg et al., 2006), and piglets are known to have poor thermoregulatory capacities at birth (postnatal hypothermia) (Kammersgaard et al., 2011). It is assumed that pigs lost UCP1 function and the ability to use BAT for thermoregulation because of absent or only weak selection for this mechanism in a warm climate, arguably because it will be energetically costly to produce large amounts of this 32 KD protein (Berg et al., 2006). All Suidae species except the wild boar, *Sus scrofa*, live only in tropical or subtropical habitats. To cope with adverse thermal conditions in northern habitats, wild boar apparently evolved compensatory mechanisms like larger adult body size (Vetter et al., 2015), building insulating nests for offspring, and synchronizing reproduction within social groups, enabling piglets to huddle in large groups of combined litters (Graves, 1984; Berg et al., 2006). Nevertheless, piglet mortality is still high and often attributed to thermoregulatory problems (Herpin et al., 2002), which could be due to the lack of BAT. However, there is evidence that while cold-induced shivering intensity decreases, simultaneously measured metabolic rate (i.e., heat production) increases (Berthon et al., 1994). This change in the ratio between shivering and metabolic rate leads to a >five-fold apparent improvement of shivering efficiency (Berthon et al., 1994). We hypothesize that the enhancement of thermogenesis in piglets is actually due to an increasing contribution of SERCA-based Ca^2+^ slippage in skeletal muscles. Importantly, this would mean that muscle NST and shivering can occur at the same time. A recent study found that in rather cold-tolerant breeds of domestic pigs, UCP3—a paralog of UCP1 found in so called beige cells, which manifested after cold exposure and showed a similar heat production potential as BAT—has a thermoregulatory function (Lin et al., 2017). However, this mechanism is not found in all pig breeds, i.e., non-detectable in cold-sensitive pigs. Blockage of shivering did not lead to a significant drop in T_b_ in cold-resistant pigs, suggesting that those breeds possess a heat production mechanism other than shivering (Lin et al., 2017). Because blocking the Ca^2+^ release through the RyR-receptors also did not change T_b_, the authors concluded that this mechanism cannot involve muscle NST via SERCA. However, muscle NST via SLN works independent of the activity of the RyR receptor (de Meis et al., 2005a). Furthermore, there is increasing evidence that UCP3 and UCP2 do not exhibit uncoupling function like UCP1 under physiological conditions (Trenker et al., 2007; Graier et al., 2008). The notion that muscle NST can indeed produce high amounts of heat and therefore may well play a role in piglet thermoregulation is supported by a pathological condition called malignant hyperthermia or porcine stress syndrome, as was already pointed out earlier by Rowland et al. (2014). Porcine stress syndrome is due to a mutation in the RyR in the SR, which leads to a massive release of Ca^2+^ into the cytoplasma, causing increased SERCA activity and heat generation (MacLennan and Phillips, 1992).

The second hypothesis suggesting that UCP1-mediated thermogenesis in BAT evolved because it enabled high T_b_ for parental care (Oelkrug et al., 2013) is challenged by the recent finding of tegu lizards (*Salvator merianae*, formerly *Tupinambis merianae*) that maintain high T_b_ during the reproductive season, despite a lack of BAT. Even during the colder night hours, when an increase of T_b_ via basking is not possible, T_b_ is maintained 5–6°C above ambient (Tattersall et al., 2016). Although not fully understood yet the observed increase in T_b_ is correlated with an increase in heart rate and suggests that heat is produced by endogenous NST (Tattersall et al., 2016). As reptiles do not possess BAT and muscle NST has been found in mammals and birds, which have evolved from reptilian ancestors, we hypothesize that these lizards also use a similar mechanism likely involving SERCA. Similarly, short beaked-echidnas lacking BAT show more stable and high T_b_ throughout incubation (Beard and Grigg, 2000; Nicol and Andersen, 2006).

An obvious question to consider with respect to muscle-NST is whether this type of thermogenesis can occur simultaneously with, or only in the absence of shivering. The biochemical mechanisms of both modes of thermogenesis do not seem to exclude either possibility. Once SLN induces slippage of Ca^2+^ from SERCA, this means Ca^2+^ ions are captured by SERCA from the sarcoplasma, which is followed by ATP cleavage and heat generation, and by the release of two Ca^2+^ back to the sarcoplasma. Hence, there is thermogenesis without actual transport of Ca^2+^ into the SR, and no requirement for Ca^2+^ release by RyR following nervous system induced sarcolemmal and T-tubule depolarization, which would lead to muscle contraction (de Meis et al., 2005a). Also, it is easy to envision that a cyclic capture and release of Ca^2+^ by SERCA at the SR membrane may not cause changes in sarcoplasmatic Ca^2+^ levels that cause muscle contractions. Gaining deeper insights into this question is certainly interesting for future research, but presently it seems that both scenarios are possible. For instance, the observations of Grigg et al. (1992) on rewarming echidnas would indicate pure muscle NST at lower T_b_s, while shivering is only occurring later. The data on piglets discussed above (Berthon et al., 1994), on the other hand, suggest that muscle NST, i.e., heat production that is not proportional to fiber contraction intensity, and shivering thermogenesis are not mutually exclusive. Indeed we see no mechanistic, biochemical reason why they should be. This raises the question whether muscle NST in fact is only possible during muscle activity. This seems unlikely, however, as Bal et al. (2012) found clear evidence for muscle NST in mice in which shivering was chemically blocked. Further, measurements of heat production in SR vesicles at a physiological temperature of 35°C also do not support this possibility (Mitidieri and de Meis, 1999). In these studies heat production was in fact maximal at Ca^2+^ concentrations similar to that found in the cytosol of a relaxed muscle fiber (1 μM) and decreased as the Ca^2+^ concentration was raised to a level similar to that found in the cytosol during muscle contraction (~10 μM). Conversely Inesi and Tadini-Buoninsegni (2014) have argued that the buildup of high concentrations of Ca^2+^ in the SR lumen, a prerequisite for slippage, is too slow to occur during a muscle relaxation and that cytosolic Ca^2+^ levels during relaxation would be too low to activate SERCA1a activity. These conclusions were based, however, on the kinetics of Ca^2+^ accumulation and SERCA1a activity in vesicles studied at 25°C, a temperature that is well-known to inhibit SERCA1a activity, at least in vesicles obtained from highly homoeothermic rabbits (de Meis et al., 2005b). Thus, we conclude that the preponderance of the current evidence indicates that muscle NST may occur both during muscle contraction and muscle relaxation, but certainly, further studies on this question seem highly desirable. Even if muscle NST may occur during shivering, it seems conceivable that constant Ca^2+^ cycling at the SERCA domain during slippage might interfere with and hamper highly coordinated rapid muscle contraction and relaxation during locomotion. Thus, especially small animals with relatively high cold loads may have difficulties to reconcile muscle NST with controlled locomotor activity and foraging. Among other factors (see below) this may be one of the selective advantages of a separate thermogenic tissue, namely BAT, which does not interfere with muscle contraction; but the degree of impairment of locomotion by muscle NST remains to be investigated. In this context, Rowland et al. (2014) have suggested that the predominance of fast-type skeletal muscles in rodents, which have high amounts of BAT, may have disfavored the use of muscle NST. This is because fast-type muscles, although they contain large amounts of SERCA1a, which should facilitate muscle NST, rely on glycolytic pathways for ATP production, whereas slow or intermediate types rely predominantly on oxidative metabolism. Therefore, Rowland et al. (2014) have argued that skeletal muscles with more oxidative fibers, where muscle-based NST would probably occur, may be favored in large mammals. However, despite their large fraction of fast-twitch fibers, even small rodents make use of muscle NST. As demonstrated by Jensen et al. (2008) enhanced muscle NST in transgenic mice, which have increased capacity for muscular fatty acid uptake, was accompanied by an increase in oxidative fibers. Further, Pant et al. (2015) recently found that the down-regulation of muscle NST in fast twitch skeletal muscles of neonatal mice could be prevented by cold acclimation. Thus, the fiber type composition of skeletal muscles in small mammals is flexible and can be adjusted to thermoregulatory requirements. Currently, there are, however, insufficient studies on a large enough range of species of different size to obtain a clear picture of the impact of body mass on muscle NST capacity.

The term endothermy is often taken to imply a pattern of homeothermic endothermy, i.e., birds and mammals that maintain a less fluctuating and fairly constant T_b_ over various conditions. However, many endothermic mammals and birds are actually abandoning homeothermy during challenging periods and undergo heterothermic phases during which they reduce T_b_ and metabolic rate in a state of torpor (Ruf and Geiser, 2015). Up to date at least 214 species of heterothermic mammals and birds have been identified (Ruf and Geiser, 2015) and it is now widely accepted that heterothermy is a plesiomorphic, ancient trait from which homeothermy has evolved (Grigg et al., 2004; Lovegrove, 2012). The mammalian ancestor was likely a small, nocturnal insectivorous animal that regularly used torpor (Luo et al., 2011; O'Leary et al., 2013). In placental heterotherms UCP1 plays an important role during rewarming from torpor (Nedergaard and Cannon, 1984). A study on UCP1-ablated mice has shown that although the lack of UCP1-mediated NST does not impair the expression of a full torpor bout (i.e., entry, maintenance, and rewarming), rewarming rates were about 50% lower and energetic costs were about 60% higher in UCP1-ablated than in wild-type individuals (Oelkrug et al., 2011). However, the mice were kept at warm conditions prior to the experiment, which prevents the cold-induced increase of muscle NST reported in later studies (Bal et al., 2016). Therefore, animals likely had to primarily rely on shivering thermogenesis and were not able to use muscle NST for rewarming. It would be interesting to see if cold acclimatization of animals prior to the experiment would lead to a differing result. Nevertheless, these data suggest that uncoupling of the proton gradient in BAT might have evolved to allow for a more rapid arousal and reduced energetic costs for rewarming, because slow rewarming increases the time spent at high metabolic rates (Oelkrug et al., 2011). If UCP1-ablated mice have lower rewarming rates this should also be the case for monotremes and marsupials. Indeed, studies report a comparatively low rewarming rate (about 50% lower than the similar sized marmot) in the monotreme echidnas (Geiser and Baudinette, 1990; Nicol et al., 2009) even though the echidnas were rewarming from about 7°C warmer minimum T_b_s, and T_b_ (as well as T_a_) is known to affect rewarming rates (Geiser et al., 1986; Geiser and Baudinette, 1987). Unfortunately, there is no comprehensive comparison between rewarming rates of marsupiala and placentalia that also take into account differences in T_a_ and T_b_. Among known heterothermic mammals, i.e., those undergoing daily torpor or hibernation, only six species of marsupials and monotremes are hibernators (18.8%) and show minimum T_b_s below 6°C (range: 1.3–5.9°C (Ruf and Geiser, 2015), whereas at least 87 (62.6%) heterothermic placental mammal species hibernate and, in contrast to marsupials and monotremes, can reduce their T_b_ to below zero degrees. These subzero T_b_s are known from at least eight species (Ruf and Geiser, 2015), e.g., −2.9°C in the arctic ground squirrel [*Urocitellus* (formely *Spermophilus) parryii*) (Barnes, 1989)]. Low tissue temperatures pose a problem because of Arrhenius effects, i.e., the cold-induced retardation of maximum enzyme activities. These Arrhenius effects may hamper, or at least significantly slow down, rewarming to euthermia. Theoretically, this problem could be overcome by local heating of a small thermogenic tissue, i.e., the autocatalysis of heat generating processes as the tissue warms itself. This is one of the properties of BAT and therefore BAT is likely not only increasing the speed of arousals from torpor, but was also important for rewarming from torpor at low T_b_s. Even if the temperatures at earth were warmer at the time of BAT evolution, animals will still have experienced daily and yearly fluctuations, similar to daily fluctuations in tropical habitats seen today. In contrast to skeletal muscle, BAT is small, mainly found between the shoulder blades and around the heart, and even in small mammals does not exceed 5% of body mass (Smith and Horwitz, 1969). Not surprisingly then, the local heating of BAT can be even detected by thermal imaging of skin (e.g., Symonds et al., 2012). In comparison, simply due to total heat capacity, using the same amount of energy for thermogenesis in skeletal muscles, which typically have a mass of 30–40% of body mass, in some species up to 50% (Hoppeler and Flück, 2002), would result in much smaller elevations of tissue temperature. Arguably then, the evolution of BAT was especially beneficial for heterothermic placental mammals, as it allowed them to tolerate lower levels of T_b_ as a result of the enormous reduction of metabolic rate during hibernation and torpor (Ruf and Geiser, 2015). This likely enhanced adaptive radiation of placental mammals and their ability to overwinter in the north-temperate and arctic zones. These are climates that are significantly colder than those inhabited by marsupials and monotremes, among which a preference for warm habitats (ranging from rainforests to deserts) is an ancestral trait (Mitchell et al., 2014; Oelkrug et al., 2015).

## How can birds be highly endothermic without bat?

If the evolution of BAT indeed facilitated the use of hibernation, the lack of BAT in birds may help to explain why there is only a single bird species known to truly hibernate (Jaeger, 1949; Woods and Brigham, 2004), although a number of birds show shallow daily torpor (Ruf and Geiser, 2015). This suggests that the bird thermoregulatory phenotype, compared with the typical small mammal, is characterized by a high degree of homeothermy, little heterothermy, and high exercise performance. Given their lack of BAT, it seems surprising then that muscle NST, or a combination of muscle NST and shivering, is sufficient to allow many birds to maintain very high (42°C) T_b_ even during cold exposure. Goldfinches will become hypothermic within a few minutes after cold exposure in summer, but can withstand temperatures of −70°C for hours in winter (Dawson and Carey, 1976). In cold-acclimated ducklings skeletal-muscles were identified as the major site of NST (Duchamp and Barre, 1993). The fact that muscle NST seems more efficient in birds might be partially related to the better insulation of feathers in comparison with mammalian hair (Aschoff, 1981). While birds can decrease their conductance enormously during cold exposure due the air trapped in the rigid feather structure, mammalian hair is softer and less suitable to trap air as an insulation barrier (McNab, 1966).

However, we suggest that there is another main reason for a lack of selective advantages of a BAT-like tissue in birds, which—to our knowledge—has never been considered before: skeletal muscles in birds already reach metabolic rates that are at least twice as high as in exercising small mammals, and can be as high as 8–18 times BMR (Butler et al., 1998; Videler, 2005). The rate-limiting step causing this difference between mammals and birds is the much greater capacity of avian skeletal muscles to take up circulating fatty acids (reviewed in Jenni-Eiermann, 2017). In contrast to mammals, endurance muscular work in birds can in fact be fueled to 95% by energy derived from lipids, and the use of this energy-dense fuel may have first evolved as an adaptation to energy-demanding flight. It seems logical then that placental mammals, possessing BAT, would much benefit from enhanced fatty acid import, compared with skeletal muscle cells. This is indeed ensured by the function of lipoprotein lipase, which, along with other enzymes, allows the massive import of fatty acids into BAT during thermogenesis at significantly higher rates than into skeletal muscle cells of placental mammals (Heldmaier et al., 1999; Townsend and Tseng, 2014). Interestingly, when the capacity of fatty acid import into muscle cells was increased by overexpression of lipoprotein lipase in transgenic mice, this improved cold resistance—independent from BAT thermogenesis—and elevated muscular fatty acid oxidation (Jensen et al., 2008). As noted by Jensen et al. (2008) this clearly reflects a shift toward “an avian phenotype”. Arguably, these differences in fuel import capacity into thermogenic tissues may well-explain the absence of BAT in birds, as well as the lower thermogenic capacity of marsupials and monotremes.

## Conclusions

In summary, it seems that muscle NST may have been an important step in the evolution of endothermy. Endothermy is clearly facilitated by increasing mitochondrial membrane surface (Else and Hulbert, 1981) and activity of the sodium-potassium pump, which is the greatest contributor to BMR (Clarke et al., 2013). However, apart from shivering, muscle NST via SERCA ATP hydrolysis was probably the first metabolic pathway in mammals solely used for thermogenesis. By comparison, UCP1-mediated NST in BAT seems to represent a mere “booster” of endothermy and is heavily employed only by small placental mammals, that is, by less than 20% of all endothermic mammal and bird species. A prominent role of skeletal muscle function in the evolution of endothermy, which is not only the activation of thermogenesis in response to cold load but also the static elevation of resting metabolic rates, would be expected by the above mentioned “aerobic capacity” model (Bennett and Ruben, 1979). Interestingly, after mixed support for this model from smaller studies over the last decades, strong evidence for the aerobic capacity model comes from a recent comprehensive phylogenetically informed study ranging from fish and amphibians to birds and mammals, which shows that there is in fact a positive correlation between maximum and resting metabolic rates in mammals, and that this pattern is a result of natural selection (Nespolo et al., 2017). These findings again point to an important role of enhanced muscle function and metabolism for the emergence of endothermy.

One of the reasons why the importance of muscle NST may have been underestimated in the past (but see, e.g., de Meis, 2001a; Grigg, 2004) is that it can be “masked” by shivering, which may well occur simultaneously (Berthon et al., 1994, 1996). There is no reason to assume that muscle NST and shivering are mutually exclusive as only part of the total SERCA activity may lead to Ca^2+^ cycling, and another part to the relaxation of myofibril contractions. In contrast, activation of UCP1-mediated NST occurs prior to the onset of shivering (Böckler and Heldmaier, 1983) which makes it easier to identify as a separate mechanism. However, even NST in BAT can also occur simultaneously with shivering thermogenesis (Böckler and Heldmaier, 1983). Arguably then, the evolution of endothermy was not characterized by switches from one to another, possibly improved, metabolic pathway. Instead it seems that increasing levels of endothermy were achieved by recruiting additional mechanisms of thermogenesis to muscular work during locomotion, including specialized shivering thermogenesis, increases in mitochondrial density and membrane leakage, increases in sodium-potassium pump activity, shifts in SERCA activity toward NST. Highly endothermic mammals living in cold environments apparently can use all of these mechanisms simultaneously.

There are several possible selective advantages to this last evolutionary step, the additional recruitment of UCP1-mediated NST. As already pointed out previously (Rowland et al., 2014) there may be a trade-off between fast muscle contraction and muscle NST caused by conflicting needs for fast glycolytic and slow oxidative muscle fibers, respectively. In addition, we suggest that in contrast to shivering thermogenesis, voluntary muscle contraction as needed for coordinated locomotion and foraging may be actually incompatible with Ca^2+^slippage. Further, we suggest that the evolution of BAT in addition to muscle NST was related to heterothermy being predominant among early endothermic mammals. This is because, in comparison with large muscles, a small dedicated thermogenic tissue such as BAT is much more suited to rapidly warm up and escape limiting Arrhenius effects of low tissue temperature during hibernation and torpor in harsh habitats. Finally, we argue that additional mechanisms for NST are not required by animals that have enhanced capacities to fuel muscle NST by high rates of fatty acid import. Such a group of endotherms are birds, which probably evolved this superior fuel transport capacity as an adaptation to flight. This would explain why birds have high endothermic capacities, despite the absence of BAT.

## Author contributions

JN wrote the first draft of the manuscript. All authors added text, discussed, and edited the manuscript.

### Conflict of interest statement

The authors declare that the research was conducted in the absence of any commercial or financial relationships that could be construed as a potential conflict of interest.

## Abbreviations

ATP: Adenosine triphosphate
BAT: Brown adipose tissue
BMR: Basal metabolic rate
NST: Non-shivering thermogenesis
PUFA: Polyunsaturated fatty acids
RyR: Ryanodine receptor
SLN: Sarcolipin
SERCA: Sarcoplasmatic reticulum Ca^2+^-ATPase
SR: Sarcoplasmatic reticulum
T_b_: Body temperature
UCP1: Uncoupling protein 1
V_max_: Maximal Velocity.
